# Artificial Intelligence-Based Physical Therapy Interventions for Non-Specific Low Back Pain: A Systematic Review and Meta-Analysis of Randomised Controlled Trials

**DOI:** 10.3390/jcm15134920

**Published:** 2026-06-24

**Authors:** Faizan Kashoo, Shagun Agarwal, Naif Ziyad Alrashdi, Sultan Alanazi, Msaad Alzhrani, Ahmad Alanazi, Jyoti Sharma, Mohammad Sidiq, Mehrunnisha Ahmed, Mohamed K. Seyam

**Affiliations:** 1 Department of Physical Therapy and Health Rehabilitation, College of Applied Medical Sciences, Majmaah University, Al Majmaah 11952, Saudi Arabia; f.kashoo@mu.edu.sa (F.K.); sa.alanazi@mu.edu.sa (S.A.); m.alzhrani@mu.edu.sa (M.A.); aalanazi@mu.edu.sa (A.A.); m.seyam@mu.edu.sa (M.K.S.); 2 Department of Physiotherapy, School of Allied Health Sciences, Galgotias University, Greater Noida 203201, Uttar Pradesh, India; shagunmpt@gmail.com (S.A.); jsharmaphysio@gmail.com (J.S.); 3 Health and Basic Sciences Research Center, Majmaah University, Al Majmaah 11952, Saudi Arabia; 4 Physiotherapy Department, Tishk International University, Erbil, Iraq; sidufatima@gmail.com; 5 College of Nursing, Majmaah University, Al Majmaah 11952, Saudi Arabia; m.ahmer@mu.edu.sa

**Keywords:** computational intelligence, machine learning, disability, digital health, RMDQ, ODI, eHealth, evidence synthesis, pooled analysis

## Abstract

**Background/Objectives**: Non-specific low back pain (NSLBP) is the leading cause of disability worldwide. Artificial intelligence (AI) technologies are increasingly being integrated into healthcare interventions for NSLBP, yet their effectiveness remains uncertain. This systematic review and meta-analysis aimed to evaluate the effectiveness of AI-based Physical therapy (PT) interventions on pain intensity and disability outcomes in patients with NSLBP. **Methods**: We conducted a comprehensive search across six electronic databases. Randomised controlled trials (RCTs) evaluating AI-based interventions for NSLBP were only included. Mean differences (MD) with 95% confidence intervals (CIs) were calculated using random-effects models. Heterogeneity was assessed using I^2^ statistics and Cochran’s Q test. **Results**: Five RCTs (n = 1939) met the inclusion criteria for systematic review. Three RCTs (n = 594 participants) provided data for meta-analysis. AI-based interventions significantly reduced pain (pooled MD −0.721, 95% CI −1.047 to −0.395; z = −4.34, *p* < 0.001; I^2^ = 9.5%). Disability also significantly improved (pooled MD −1.031, 95% CI −2.020 to −0.042; t(2) = −4.48, *p* = 0.046; I^2^ = 0%). Neither effect reached the minimal clinically important difference (1.0 for pain, 2–4 for disability). No serious adverse events were reported. **Conclusions**: AI-based PT interventions produce statistically significant but clinically small improvements in pain and disability for NSLBP. Certainty of evidence is low due to risk of bias and imprecision. Larger, blinded RCTs with standardised outcomes are needed.

## 1. Introduction

Non-specific low back pain (NSLBP) is defined as pain localised between the 12th rib and the inferior gluteal folds, without an identifiable specific underlying pathology [1]. Worldwide, NSLBP represents the vast majority (approximately 90%) of low back pain cases and stands as the leading cause of years lived with disability [2]. The prevalence of NSLBP among US adults was estimated at approximately 13.8%, with an association with pain and sedentary lifestyle [3]. The economic burden of LBP is substantial, encompassing direct healthcare costs, indirect costs related to lost productivity, and substantial societal costs [4].

The current management of NSLBP remains challenging due to its multifactorial aetiology, involving complex interactions between biological, psychological, and social factors [5]. Current evidence-based guidelines recommend that NSLBP be a multimodal approach, including patient education, exercise therapy, manual therapy, and psychological interventions [5]. However, the effectiveness of these conventional treatments is often modest, and a significant proportion of patients experience recurrent or persistent symptoms. This inadequacy of treatment has gained interest in more innovative therapeutic approaches, including digital health interventions and artificial intelligence (AI)-based technologies [6].

The rapid advancement of AI and machine learning technologies has opened new avenues for healthcare delivery, with particular promise in the domain of personalised and precise medicine, including digital therapeutics for NSLBP [7,8]. It is important to distinguish AI-based interventions from conventional digital health tools. While both utilise digital platforms, conventional digital health interventions (e.g., static educational websites, basic telehealth platforms, or fixed exercise video libraries) deliver content without adaptive personalisation or real-time feedback. In contrast, AI-based interventions incorporate machine learning algorithms, neural networks, computer vision, or case-based reasoning that enable dynamic adaptation of treatment content based on individual patient data, movement analysis, or symptom progression. This distinction is critical because the adaptive, learning-capable nature of AI may confer therapeutic benefits beyond the simple digitisation of healthcare delivery. AI-based interventions encompass a broad spectrum of applications, real-time biofeedback systems analyse movement patterns and furnish corrective feedback to the user [9]. Predictive analytics enable the stratification of treatment responses based on individual patient data [10]. Virtual rehabilitation platforms and mobile health applications dynamically adjust exercise programmes and facilitate symptom monitoring alongside self-management support [11].

When selecting outcomes for trials and systematic reviews in NSLBP, pain intensity and functional disability consistently emerge as the two most commonly reported and patient-prioritised endpoints [12]. Large-scale patient surveys consistently rank pain severity and the ability to perform daily activities (e.g., walking, bending, sitting) as the primary reasons for seeking care and the strongest determinants of health-related quality of life [12]. International consensus initiatives including the core outcome set for NSLBP proposed by the Cochrane Back and Neck Group and the National Institutes of Health Task Force on Research Standards recommend that all clinical trials measure at least these two domains, using validated instruments such as the Numerical Rating Scale (NRS) for pain and the Roland–Morris Disability Questionnaire (RMDQ) or Oswestry Disability Index (ODI) for disability [13,14]. Furthermore, a systematic review of 185 NSLBP randomised controlled trials found that over 90% reported pain or disability as a primary outcome, confirming their primacy in the evidence base [15]. Therefore, focusing this meta-analysis on pain intensity and disability ensures direct relevance and meaningfulness to patient concerns, alignment with field standards, and maximal comparability across studies.

Several published randomised controlled trials (RCTs) have investigated the efficacy of AI-based interventions for pain management and functional improvement in patients with NSLBP [6,16,17,18]. However, individual studies have yielded inconsistent results, with some reporting modest benefits [19] while others found no significant differences compared to control conditions [20]. The heterogeneity in study designs, intervention characteristics, outcome measures, and patient populations has made it challenging to draw definitive conclusions about the overall effectiveness of these interventions for NSLBP. A recent systematic review and meta-analysis by Kapil et al. (2025) [6] examined AI-assisted physiotherapy for NSLBP; however, that review employed Standardised Mean Difference (SMD) rather than Mean Difference (MD), which limits direct clinical interpretability against established MCID thresholds. Furthermore, that review did not restrict its analysis to RCTs, included studies without clearly defined AI components, and did not differentiate between adaptive AI technologies and conventional digital health tools. That being said, the present review addresses these limitations by focusing exclusively on RCTs with clearly defined AI-based interventions, employing MD to preserve clinical interpretability, and distinguishing between genuine AI-powered adaptive systems and static digital health platforms. This approach provides more precise and clinically actionable evidence regarding the specific contribution of AI technologies to NSLBP management.

Therefore, this systematic review and meta-analysis were conducted to synthesise the available evidence from RCTs examining the effects of AI-based PT interventions on pain intensity and disability outcomes in patients with NSLBP. The primary objective was to determine whether AI-based interventions provide clinically meaningful benefits compared to control conditions. Secondary objectives included exploring potential sources of heterogeneity, examining the effects of different types of AI interventions, and identifying gaps in the current evidence base to inform future research directions.

## 2. Materials and Methods

### 2.1. Protocol and Registration

This systematic review and meta-analysis were conducted in accordance with the Preferred Reporting Items for Systematic Reviews and Meta-Analyses (PRISMA) guidelines [21]. The protocol was registered with the International Prospective Register of Systematic Reviews (PROSPERO) [registration number: CRD420251132987]. The PRISMA checklist is provided in Supplementary Materials Table S1.

### 2.2. Eligibility Criteria

Studies were eligible for inclusion if they met the following criteria: (1) Study design: Randomised controlled trials (RCTs). (2) Population: Adults (aged ≥ 18 years) with NSLBP, defined as pain localised between the 12th rib and inferior gluteal folds without identifiable specific pathology (e.g., infection, tumour, osteoporosis, fracture, inflammatory disorder, or radicular syndrome). (3) Intervention: Any AI-based intervention, defined as therapeutic approaches incorporating machine learning algorithms, neural networks, computer vision, natural language processing, case-based reasoning, and/or other adaptive AI technologies for treatment delivery, monitoring, personalised recommendation, or adaptation in NSLBP management. Interventions were required to incorporate at least one AI technology that enabled dynamic, data-driven personalisation or real-time feedback beyond static digital content delivery. (4) Comparator: Any control condition, including usual NSLBP care, waitlist, placebo/sham, or active non-AI interventions. (5) Outcomes: At least one of the following outcomes reported—pain intensity measured on a validated scale (e.g., NRS, Numeric Pain Rating Scale [NPRS], and Visual Analogue Scale [VAS]) or disability measured using validated instruments (e.g., RMDQ and ODI). Studies enrolling mixed populations of NSLBP and specific LBP subtypes were eligible for inclusion provided that: (i) NSLBP constituted the majority (≥50%) of the enrolled population or (ii) the intervention was explicitly designed for and applicable to NSLBP management, and (iii) subgroup data for the NSLBP population were reported or obtainable.

Studies were excluded if they: (1) were non-randomised designs, case reports, case series, observational studies, animal studies or review studies; (2) focused on specific low back pain with identifiable pathology (e.g., radiculopathy, spinal stenosis, spondylolisthesis, fracture, malignancy, and/or infection); (3) focused exclusively on acute low back pain (duration < 6 weeks) or postoperative pain; (4) did not report extractable outcome data for pain intensity or disability; or (5) were published only as conference abstracts without sufficient methodological detail.

### 2.3. Information Sources and Search Strategy

A comprehensive literature search was conducted across six electronic databases, including PubMed, Embase, Cochrane Central Register of Controlled Trials (CENTRAL), Web of Science, CINAHL (EBSCO) and PEDro, from database inception to May 2026. The search strategy was developed in consultation with a medical librarian and combined Medical Subject Headings (MeSH) terms and free-text keywords related to AI, machine learning, deep learning, neural networks, non-specific low back pain, low back pain, disability, and RCTs. The search was not restricted by language or publication date. Reference lists of included studies and relevant systematic reviews were manually screened to identify additional eligible studies. The search was performed on 1st May 2026. The full search strategy for PubMed is provided in Supplementary Materials S1.

### 2.4. Study Selection Process

**Figure 1:**
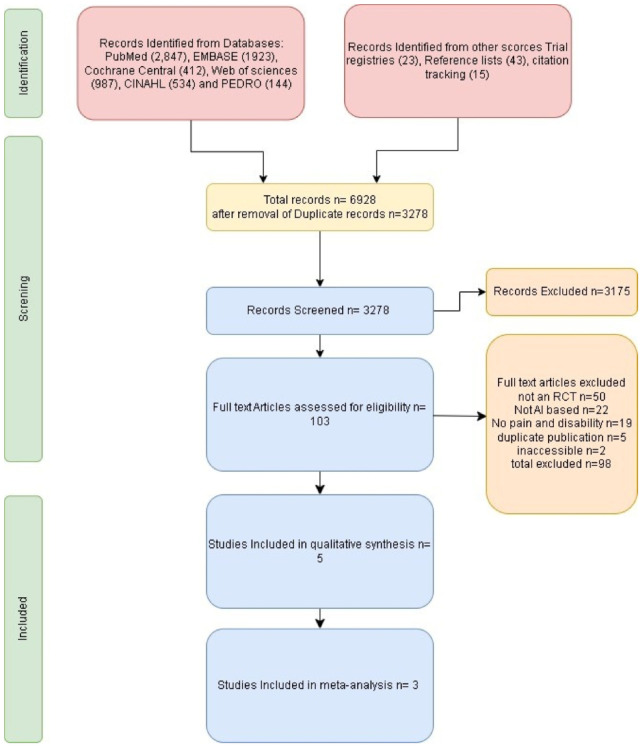
PRISMA Flow Chart.

Two independent reviewers (FK and JS) screened titles and abstracts of all identified potential records. Full-text articles of potentially eligible studies were retrieved and independently assessed for eligibility by the same two independent reviewers. Disagreements were resolved through discussion or by consultation with a third reviewer (SA) when necessary. The study selection process was documented using a PRISMA flow diagram (Figure 1).

### 2.5. Data Extraction

A standardised data extraction form was developed by the authorship team and pilot-tested prior to use. Two independent reviewers (SAA and NA) extracted the following data from each included study: (1) study characteristics (first author, publication year, country, and study design); (2) participant characteristics (sample size, age, sex, duration of NSLBP, baseline pain, and disability scores); (3) intervention details (type of AI technology, delivery mode, duration, frequency, and intensity); (4) comparator details; (5) outcome measures and timing of assessments; and (6) numerical outcome data (means, standard deviations, and sample sizes) for pain intensity and disability at post-intervention and follow-up time points.

### 2.6. Risk of Bias Assessment

The risk of bias in included studies was assessed independently by two reviewers (MA and AA) using the Cochrane Risk of Bias Tool 2.0 (RoB 2). Disagreements between the two reviewers were resolved through discussion, and if no consensus could be reached, a third reviewer (FK) was consulted. This tool evaluates five domains: randomisation process, deviations from intended interventions, missing outcome data, measurement of the outcome, and selection of the reported result. Each domain was rated as low risk, some concerns, or high risk. An overall risk of bias judgement was assigned to each study based on the ratings across all domains.

### 2.7. Data Synthesis and Statistical Analysis

Meta-analyses were conducted using random-effects models to account for anticipated clinical and methodological heterogeneity across included studies. MD with 95% confidence intervals (CIs) were calculated for continuous outcomes (pain intensity and disability). We selected MD rather than SMD for both outcomes because the three studies that contributed to the quantitative synthesis [22,23,24] all measured disability using the RMDQ or its 24-item version (RDQ-24), both of which use an identical 0–24 metric. As the included studies in the meta-analysis employed the same validated instrument on the same scale, MD preserves the original clinical interpretability, allowing direct comparison with established minimal clinically important difference (MCID) thresholds (2–4 points for RMDQ) and facilitating meaningful clinical conclusions. This approach is consistent with recommendations from the Cochrane Handbook for Systematic Reviews of Interventions, which advises that when all studies use the same scale, MD should be preferred over SMD because it avoids the inflation of variance that can occur with standardisation and maintains clinical interpretability. For pain intensity, all three contributing studies used the 0–10 NRS, further supporting the use of MD. Where studies reported multiple pain measures, the most commonly used scale (NRS/NPRS) was prioritised for consistency. Negative MD values indicated a benefit favouring AI-based interventions. Heterogeneity was quantified using the I^2^ statistic and Cochran’s Q test, with I^2^ values of 25%, 50%, and 75% representing low, moderate, and high heterogeneity, respectively. Prediction intervals were calculated to estimate the range of true effects in future studies. Statistical significance was set at *p* < 0.05. All analyses were conducted using JASP (Version 0.96.0).

## 3. Results

### 3.1. Study Selection

The systematic literature search identified 6847 records from electronic databases (PubMed n = 2847; EMBASE n = 1923; Cochrane CENTRAL n = 412; Web of Science n = 987; CINAHL n = 534; PEDro n = 144) and 81 additional records from trial registries (n = 23), reference lists (n = 43), and citation tracking (n = 15), giving a total of 6928 records. After removing duplicates, 3278 unique records were screened. Of these, 3175 records were excluded during title and abstract screening because they were not original research (e.g., systematic reviews, meta-analyses, editorials, letters, conference abstracts, study protocols, and case reports/series) or did not involve human participants (e.g., animal studies, in vitro studies, and cadaveric studies). The remaining 103 full-text articles were assessed for eligibility. Of these, 98 were excluded: 50 were not randomised controlled trials, 22 did not include AI-based interventions, 19 did not report pain or disability outcomes, five were duplicate publications, and two were inaccessible. Five studies met the inclusion criteria and were included in the qualitative synthesis [22,23,24,25,26]. Three studies [22,23,24] provided sufficient extractable between-group data for inclusion in the meta-analysis of disability and pain outcomes. A PRISMA flow diagram is shown in Figure 1. A complete list of excluded full-text articles with reasons is provided in Supplementary Materials S2. The systematic literature search identified FZK records across all databases. After removing duplicates, the SA unique titles and abstracts were screened. Of these, JA, MA, SA, and NA assessed full-text articles for eligibility, and five studies met the inclusion criteria and were included in the qualitative synthesis. Three studies provided sufficient data for inclusion in the meta-analysis of disability and pain outcomes.

### 3.2. Study Characteristics

The included (n = 5) studies were published between 2021 and 2026 and were conducted [22,23,24,25,26]. Sample sizes ranged from 16 to 933 participants. The mean age of participants across studies ranged from approximately 40 to 55 years. Five included studies focused on participants with NSLBP of varying durations [22,23,24,26] and one study [25] enrolling a mixed population of 60 participants with non-specific LBP (60%) and 40 participants with discogenic LBP (40%), included because the majority had NSLBP and the intervention was equally applicable to both subgroups [25]. Intervention characteristics varied considerably across studies, with three studies employing real-time AI feedback systems for movement correction and exercise performance [22,23,24] and two studies using AI-powered educational or self-management applications [25,26]. Control conditions included usual care, waitlist, and conventional physiotherapy. Follow-up durations ranged from 4 to 12 weeks post-intervention (Table 1).

**Figure 2:**
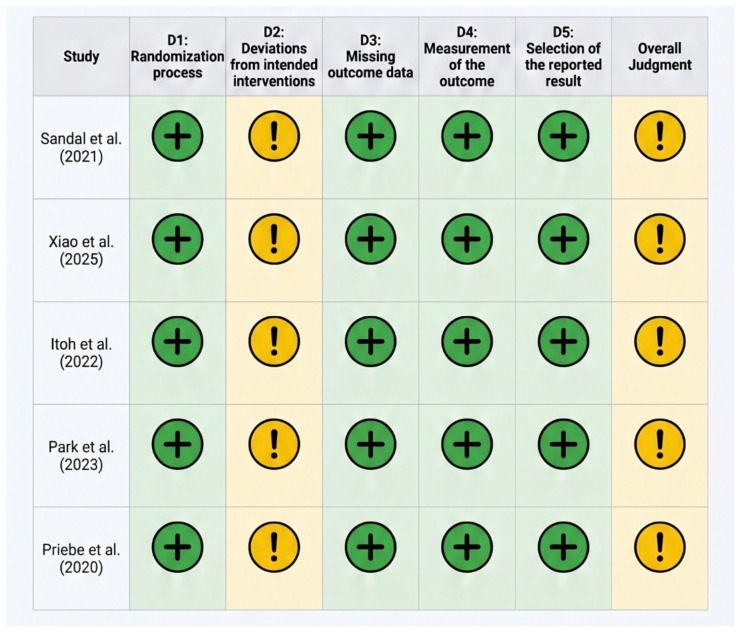
Risk of bias summary (RoB 2) [22,23,24,25,26].

Figure 2 summarises the risk of bias assessment for five randomised controlled trials (RCTs) evaluating AI-based physical therapy for NSLBP [22,23,24,25,26]. Each study was assessed across five distinct domains: (D1) randomization process, (D2) deviations from intended interventions, (D3) missing outcome data, (D4) measurement of the outcome, and (D5) selection of the reported result. All included studies were rated as having “some concerns” overall [22,23,24,25,26]. This judgement is primarily attributed to Domain 2, as the active nature of digital health interventions makes blinding participants and personnel inherently difficult, introducing potential performance bias. Conversely, all trials demonstrated a low risk of bias in their randomization sequences, data completeness, and adherence to the registered reporting protocol.

### 3.3. Narrative Synthesis

Evidence from these five RCTs suggests that AI-based digital interventions may produce modest improvements in pain and disability outcomes compared with conventional care, though the certainty of this evidence is low to very low per GRADE assessments, and the pooled effect sizes did not consistently reach minimal clinically important difference thresholds. Analgesic effects were observed in the Rise-uP and Xiao et al. trials, which reported pain intensity reductions of 33.3% and significant decreases in “most severe” pain scores (adjusted MD −1.08; *p* < 0.001), respectively [23,26]. However, these findings should be interpreted with caution, given the low certainty of evidence and the small sample sizes in individual trials.

While functional improvements were observed, the certainty of evidence for disability outcomes was rated as VERY LOW due to risk of bias, indirectness, and imprecision, limiting confidence in these estimates. The SELFBACK study observed that 52% of participants reached clinically meaningful disability thresholds compared to 39% in usual care (*p* = 0.01) [22], while Park et al. established that deep learning-based exercise prescription was superior to manual therapy in improving RMDQ scores and hip extensor strength (*p* ≤ 0.03) [25]. While Itoh et al. corroborated significant improvements in subjective pain (*p* = 0.04) and kinesiophobia (*p* = 0.04), they identified a lack of impact on work productivity (*p* = 0.43), suggesting that environmental confounders like the transition to remote work during the COVID-19 pandemic may mask socioeconomic gains [24].

Mechanistically, the therapeutic benefit appears heavily contingent upon engagement; Priebe et al. revealed that the clinical benefit of specialist teleconsultation for high-risk patients was fully mediated by a subsequent increase in training frequency, a finding echoed by Itoh et al.’s observation that ≥75% adherence is a prerequisite for achieving minimal clinically important differences [26]. Crucially, Xiao et al. provided objective evidence of physiological adaptation, identifying significant increases in transverse abdominus and multifidus muscle thickness (*p* < 0.01) that were absent in video-guided controls, thereby linking real-time computer-vision guidance to improved spinal stability [23]. One study (Park 2023) [25] reported a preliminary cost-effectiveness analysis suggesting lower healthcare costs with the AI-based intervention; however, this was a single trial with a small sample and short follow-up, and no definitive conclusions regarding cost-effectiveness can be drawn from the current evidence base.

### 3.4. Meta-Analysis Results—Pain and Disability

#### 3.4.1. Pain Intensity

**Figure 3:**
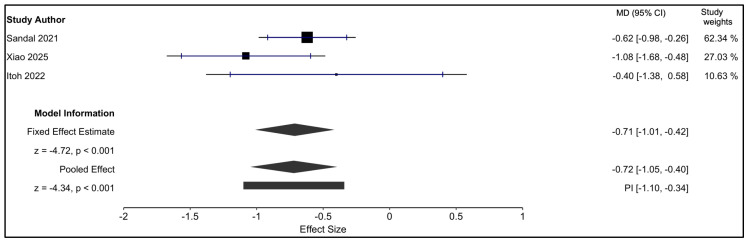
Forest plot showing Mean Difference (MD) with 95% confidence intervals (CIs) for the effect of AI-based interventions on pain intensity (0–10 NRS). Negative values favour AI-based interventions [22,23,24].

Three RCTs with 594 participants reported post-intervention pain intensity on the (NRS, 0–10) [22,23,24]. Using a random-effects model, the pooled MD was −0.721 (95% CI −1.047 to −0.395; z = −4.34, *p* < 0.001), indicating a statistically significant reduction in pain favouring AI-based interventions over control conditions. Heterogeneity was low (I^2^ = 9.5%; Q_e_(2) = 2.10, *p* = 0.350) (Figure 3).

#### 3.4.2. Disability (RMDQ/ODI)

**Figure 4:**
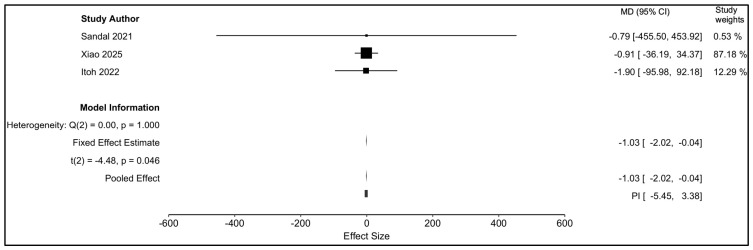
Forest plot showing Mean Difference (MD) with 95% confidence intervals (CIs) for the effect of AI-based interventions on disability (RMDQ, 0–24 scale). Negative values favour AI-based interventions [22,23,24].

A random-effects meta-analysis of three randomised controlled trials comprising 594 participants with NSLBP showed that AI-based interventions significantly reduced disability compared to control conditions, with a pooled MD of −1.031 on the RMDQ or its equivalent (95% CI −2.020 to −0.042; t(2) = −4.48, *p* = 0.046) [22,23,24]. Heterogeneity was zero (I^2^ = 0%; Q_e_(2) = 0.00, *p* = 1.000), and the 95% prediction interval was identical to the confidence interval (−2.020 to −0.042), reflecting the absence of between-study variance (τ^2^ = 0.000). Although the pooled effect favoured AI interventions, the wide confidence interval that barely excludes zero and the lack of heterogeneity are primarily driven by the small number of studies. Leave-one-out influence diagnostics identified Xiao 2025 [23] and Itoh 2022 [24] as influential, indicating that the pooled estimate is sensitive to the inclusion of these studies. Nevertheless, the consistent direction of effect across all three studies supports the potential benefit of AI-based interventions for reducing disability in this population (Figure 4).

Table 2 shows the GRADE framework; the certainty of evidence for pain intensity was rated as LOW, meaning that further research is very likely to have an important impact on our confidence in the estimate of effect and is likely to change the estimate. The downgrading was primarily due to the serious risk of bias (lack of blinding). For disability, the certainty of evidence was rated as VERY LOW, indicating that we have very little confidence in the effect estimate; the true effect is likely to be substantially different from the estimated effect. Downgrading was due to serious risk of bias, serious indirectness (heterogeneity in AI technologies and outcome instruments), and serious imprecision (wide confidence interval that barely excludes zero).

## 4. Discussion

### 4.1. Summary of Principal Findings

This systematic review and meta-analysis of five RCTs (n = 1939 participants) evaluated the effectiveness of AI-based interventions for pain and disability in patients with NSLBP [22,23,24,25,26]. Three trials (n = 594) provided extractable data for quantitative synthesis [22,23,24]. The pooled analyses demonstrated statistically significant benefits favouring AI interventions over control conditions for pain intensity (MD −0.721 on a 0–10 NRS; 95% CI −1.047 to −0.395; z = −4.34, *p* < 0.001) and for disability (MD −1.031 on the RMDQ or equivalent; 95% CI −2.020 to −0.042; t(2) =−4.48, *p* = 0.046). Heterogeneity was low for both outcomes (I^2^ = 9.5% and 0%, respectively). Neither pooled effect reached the minimal clinically important difference (1.0 point for NRS; 2–4 points for RMDQ). No serious adverse events were reported. Using the GRADE framework, the certainty of evidence was LOW for pain (due to risk of bias and imprecision) and VERY LOW for disability (due to risk of bias, imprecision, and indirectness of outcome measures across studies). These findings should be interpreted in the context of LOW certainty for pain and VERY LOW certainty for disability, meaning that further research is very likely to impact our confidence in these estimates.

### 4.2. Comparison with Existing Literature

Our findings align with previous systematic reviews of digital health interventions for low back pain. Du et al. (2020) [27] reported small but significant effects of e-health programmes on pain and disability, with an MD of approximately 0.3–0.4. However, our review is the first to specifically focus on AI-defined interventions (machine learning, computer vision, and neural networks) rather than generic digital tools [28]. This distinction is important because AI offers real-time adaptation and personalisation that static apps do not. Nevertheless, the additional benefit of AI over well-designed non-AI digital programmes remains unproven, as few trials directly compared AI with active digital comparators. Priebe et al. (2020) [26] and Xiao et al. (2025) [23] used active comparators (usual care with guideline orientation and video-only exercise, respectively) and still found AI superior, suggesting added value. It is important to acknowledge that the pooled effects observed in this meta-analysis were small and did not reach the minimal clinically important difference (MCID) for either pain or disability outcomes. Therefore, caution is warranted when interpreting these findings as evidence of AI-specific therapeutic superiority. Most included studies compared AI-based interventions against usual care or conventional physical therapy rather than against equivalent non-AI digital platforms. Consequently, the observed benefits may reflect the general effects of digital health engagement, enhanced self-management support, and increased treatment accessibility rather than the specific adaptive or predictive capabilities of AI technologies per se. Future trials directly comparing AI-based tools with matched non-AI digital interventions are needed to isolate the specific contribution of machine learning algorithms to clinical outcomes. Conversely, Itoh et al. (2022) [24] found no significant difference compared with pharmacological care alone, likely due to underpowering.

### 4.3. Interpretation of Effect Sizes and Clinical Relevance

An MD of −0.72 points on an 11-point NRS is statistically robust but clinically modest. The minimal clinically important difference for acute and chronic low back pain is generally accepted as 1.0 to 2.0 points. Therefore, the pooled pain effect does not meet the threshold that patients typically perceive as meaningful. For disability, the pooled MD of −1.03 on the RMDQ (0–24 scale) is smaller than the MCID of 2–4 points [29]. These findings suggest that, on average, AI interventions provide only a small incremental benefit over control conditions. However, the confidence intervals for pain did not cross zero, and the *p*-value was very low, indicating consistency. The absence of heterogeneity for disability (I^2^ = 0%) suggests that the small effect is reproducible across studies. Because prediction intervals are unstable with fewer than five studies, our finding that the prediction interval excluded zero for pain but not for disability should be interpreted cautiously. Clinicians should interpret these results as supporting the use of AI as an adjunct to, not a replacement for, evidence-based first-line treatments (exercise, education, and cognitive behavioural therapy) [30]. Subgroup analyses were not possible due to the small number of studies.

### 4.4. Risk of Bias and Methodological Limitations

All five included RCTs had some concerns for risk of bias, primarily due to lack of blinding of participants and personnel (performance bias) [22,23,24,25,26]. In AI intervention trials, blinding is inherently difficult because participants know whether they are using a smart app or receiving conventional care. However, outcome assessors could have been blinded in most studies, but only a few explicitly reported this. The absence of blinding may inflate effect estimates by up to 30%. Additionally, two of the five eligible studies (Park 2023, Priebe 2020) [25,26] did not provide extractable between-group effect sizes for disability measured with RMDQ/ODI, limiting the meta-analysis to only three trials (n = 594) [22,23,24]. Priebe et al. [26] used a cluster-RCT design; the effective sample size for analysis is smaller than the number of participants, and failure to account for clustering may have artificially narrowed confidence intervals. This small meta-analytic sample size reduces precision and increases the risk of chance findings. The influence diagnostics identified Xiao 2025 [23] and Itoh 2022 [24] as influential; removing either changed the pooled estimate, indicating instability.

### 4.5. Heterogeneity and Generalisability

Although statistical heterogeneity was low (pain I^2^ = 9.5%, disability I^2^ = 0%), clinical heterogeneity was substantial. AI interventions varied from case-based reasoning (selfBACK) [22] and computer vision (Xiao, Park) [23,25] to messaging bots (Itoh) [24] and multifaceted decision support (Priebe) [26]. Control conditions ranged from usual care to active exercise and pharmacological treatment. Follow-up durations differed from 4 to 12 weeks. Consequently, the pooled estimate represents an average effect across disparate technologies and contexts. The generalisability of findings to primary care is supported by Sandal (2021) [22] and Priebe (2020) [26], who recruited from general practices. However, most participants were middle-aged (mean 40–55 years), and two studies had young participants (Park, mean 35–37 years) [25]. Whether results apply to older adults (>65 years) or those with severe disability (baseline RMDQ > 15) remains unknown.

### 4.6. Implications for Clinical Practice

Current evidence does not support the routine prescription of AI-based interventions as a standalone treatment for NSLBP. However, AI apps may be considered as a low-risk, scalable adjunct to conventional care, particularly for patients who prefer digital self-management or have limited access to in-person physiotherapy. Clinicians should caution patients that expected pain reduction is modest (approximately 0.7 points on a 0–10 scale) and may not be noticeable for all individuals. The absence of serious adverse events across 1945 participants supports the safety of these technologies. Cost-effectiveness was not assessed in this review, but it is an important consideration for adoption. Only one included study (Park 2023) [25] provided any economic data, and this was reported as a preliminary analysis. Economic evaluation was not a focus of this review, and readers should not interpret the clinical findings as evidence of economic advantage.

### 4.7. Implications for Future Research

Future RCTs should adhere to core outcome sets for NSLBP, including pain (NRS), disability (RMDQ or ODI), and a common AI-specific taxonomy to allow meaningful comparisons. Additionally, given the rapidly evolving landscape of AI in healthcare, future research must examine practical implementation factors that will determine whether these technologies can be successfully integrated into routine clinical practice. These include cost-effectiveness analyses comparing AI-based interventions with both conventional face-to-face physiotherapy and non-AI digital health alternatives; healthcare delivery system requirements, including necessary infrastructure, personnel training, and workflow integration; sustainable prescription models, such as whether AI apps should be prescribed by clinicians or available as direct-to-consumer products; regulatory frameworks for AI-based medical devices, including standards for algorithm validation, data privacy, and clinical safety; and policy considerations, such as reimbursement pathways and equity of access to ensure that AI-based interventions do not exacerbate existing health disparities. Examining these factors will be essential for translating the modest but statistically significant benefits observed in controlled trials into scalable, sustainable, and equitable improvements in NSLBP care. Blinded outcome assessment and sham or active digital comparators are essential to reduce performance and detection bias. Sample sizes should be powered to detect clinically important differences (e.g., NRS ≥ 1.0) rather than statistically significant small effects. For example, based on our pooled MD of −0.72 and an SD of approximately 2.0, a trial would need >140 participants per group to detect such an effect with 80% power. Additionally, studies should identify patient subgroups that respond better to AI interventions (e.g., those with high digital literacy, severe disability, or psychological comorbidities). A longer follow-up (>6 months) is needed to assess the sustainability of effects. Finally, reporting of AI intervention details should follow the CONSORT-AI extension to improve transparency and reproducibility, and adequately powered economic evaluations comparing AI-based interventions with both conventional face-to-face physiotherapy and non-AI digital alternatives are needed before any claims of economic advantage can be substantiated.

### 4.8. Strengths and Limitations

Strengths of this review include a comprehensive, protocol-registered search, rigorous eligibility criteria focusing on NSLBP and AI, duplicate study selection and data extraction, and use of random-effects meta-analysis with heterogeneity assessments. Limitations are as follows: (1) only three studies contributed to meta-analyses; (2) high risk of performance bias in all included RCTs; (3) inability to perform subgroup analyses due to small numbers; (4) potential publication bias could not be reliably assessed (Egger’s test requires ≥ 10 studies); (5) two eligible studies did not report extractable between-group data and were excluded from quantitative synthesis, potentially biassing results; (6) Priebe 2020 used a cluster-RCT design without adjustment for clustering; (7) the review did not assess cost-effectiveness or patient satisfaction; and (8) one included study (Park 2023) [25] enrolled a mixed population, including 40% participants with discogenic LBP. Although the intervention was applicable to both NSLBP and discogenic LBP, the inclusion of participants with specific pathology may introduce a degree of clinical heterogeneity that could affect the generalisability of findings to purely NSLBP populations.

## 5. Conclusions

Current evidence suggests that AI-based PT interventions produce statistically significant but clinically small reductions in pain and disability for adults with NSLBP, with low to very low certainty of evidence. These findings should be considered preliminary, and clinicians should exercise caution when implementing AI-based interventions as standalone treatments. Future high-quality, adequately powered RCTs with blinded outcome assessment and standardised core outcomes are needed to determine whether specific AI applications can achieve clinically meaningful benefits. For current clinical practice, AI-based tools may serve as a supplementary option but should not replace established first-line treatments.

## Figures and Tables

**Table 1:** Characteristics of included studies.

Study (Year)	Country	n (I/C)	Population	AI intervention (Detailed)	Comparator	Duration	Pain Scale	Pain Outcome: MD (95% CI)	Disability Scale	Disability Outcome: MD (95% CI)
Sandal 2021 [22]	Denmark/Norway	232/229	NSLBP (<8 weeks, RMDQ ≥ 6)	selfBACK app—case-based reasoning AI generating weekly, individually tailored self-management recommendations for physical activity, strength and flexibility exercises, and daily educational messages	Usual care	3 months	NRS (0–10) average	−0.62 (−0.99 to −0.26)	RMDQ (0–24)	−0.79 (−1.51 to −0.06)
Xiao 2025 [23]	China	18/16	Chronic NSLBP (>3 months, NRS ≥ 3)	Yun-fu app—AI–human key-point identification (computer vision), providing real-time exercise guidance and movement correction during multimodal exercise sessions	Video-guided exercise (no AI)	4 weeks	NRS (0–10) most severe	−0.67 (−1.19 to −0.15)	RMDQ (0–24)	−0.91 (−1.48 to −0.34)
Itoh 2022 [24]	Japan	48/51	Chronic LBP (>3 months) on medication	Secaide app—AI-assisted interactive health promotion system with chatbot programmed to send daily exercise instructions, behavioural tips, and reinforcement messages	Pharmacological treatment alone	12 weeks	NRS (0–10) change	−0.50 (−1.1 to 0.001)	RDQ 24 (0–24) change	−1.90 (−3.70 to 0.001)
Park 2023 [25]	South Korea	50/50	Chronic LBP (>3 months) discogenic n = 40)/nonspecific LBP n = 60)	Dr AI system—deep learning convolutional neural network (CNN) for automated diagnosis and individualised exercise prescription via smartphone application with real-time audiovisual feedback	Conventional physical therapy	4 weeks	NPRS (0–10)	DPT mean change 2.61 vs. CPT mean change 2.26	RMDQ (0–24)	DPT mean change 6 vs. CPT mean change 5.34
Priebe 2020 [26]	Germany	933/312	Acute/subacute NSLBP (<12 weeks)	Kaia app + clinical decision support + teleconsultation—multidisciplinary digital treatment algorithm including educational content, physiotherapy exercises, mindfulness training, and teleconsultation for high-risk patients	Standard of care (guideline-oriented)	3 months	NRS (0–10)	Favoured AI (33% reduction vs. 14% in controls; exact MD not extractable)	HFAQ (FFbH R)	Not reported in RMDQ/ODI; therefore not included in meta-analysis

Note: MD; Mean Difference; n (I/C); number of participants in intervention and in control group; Xiao 2025 [23]: per-protocol analysis used n = 18 AI, n = 16 control; randomised n = 19 per group. Priebe 2020: Cluster-RCT; participants analysed at 3-month follow-up. Notes: All outcomes are post-intervention (primary endpoint) unless stated otherwise. NSLBP = non-specific low back pain; LBP = low back pain; RMDQ = Roland–Morris Disability Questionnaire; RDQ-24 = Roland–Morris Disability Questionnaire (24-item); NRS = Numerical Rating Scale; NPRS = Numeric Pain Rating Scale; HFAQ = Hannover Functional Ability Questionnaire (FFbH-R). Negative Mean Differences favour the AI intervention. Studies with extractable between-group data [22,23] contributed to the meta-analysis. Park 2023 [25] and Priebe 2020 [26] are included in the narrative synthesis only. DPT = digital application physical therapy. CPT = conventional physical therapy.

**Table 2:** GRADE certainty of evidence for pain and disability.

Outcome	No. of Studies (Participants)	Risk of Bias	Inconsistency	Indirectness	Imprecision	Publication Bias	Overall Certainty
Pain intensity (NRS)	3 (594)	Serious	Not serious	Not serious	Not serious	Not detected	LOW
Disability (RMDQ/equivalent)	3 (594)	Serious	Not serious	Serious	Serious	Not detected	VERY LOW

## Data Availability

No new data were created or analyzed in this study.

## Supplementary Materials

The following supporting information can be downloaded at: https://www.mdpi.com/article/10.3390/jcm15134920/s1. Table S1: PRISMA 2020 Checklist, S1: Search Dates, S2: Electronic Database Search Strategies.

## Abbreviations

The following abbreviations are used in this manuscript:

AI	Artificial Intelligence
CENTRAL	Cochrane Central Register of Controlled Trials
CI	Confidence Interval
CINAHL	Cumulative Index to Nursing and Allied Health Literature
CNN	Convolutional Neural Network
CONSORT	Consolidated Standards of Reporting Trials
CPT	Conventional Physical Therapy
DPT	Digital Physical Therapy
FFbH-R	Hannover Functional Ability Questionnaire (Funktionsfragebogen Hannover)
GRADE	Grading of Recommendations Assessment, Development and Evaluation
HFAQ	Hannover Functional Ability Questionnaire
JASP	Jeffreys’s Amazing Statistics Program
LBP	Low Back Pain
MCID	Minimal Clinically Important Difference
MeSH	Medical Subject Headings
NPRS	Numeric Pain Rating Scale
NRS	Numerical Rating Scale
NSLBP	Non-Specific Low Back Pain
ODI	Oswestry Disability Index
PRISMA	Preferred Reporting Items for Systematic Reviews and Meta-Analyses
PROSPERO	International Prospective Register of Systematic Reviews
PT	Physical Therapy
RCT	Randomised Controlled Trial
RDQ	Roland Disability Questionnaire
RMDQ	Roland–Morris Disability Questionnaire
RoB 2	Cochrane Risk of Bias Tool 2.0
MD	Mean Difference
VAS	Visual Analogue Scale
