# Supplementary material for: Artificial Intelligence-Based Physical Therapy Interventions for Non-Specific Low Back Pain: A Systematic Review and Meta-Analysis of Randomised Controlled Trials
Source: J Clin Med. 2026 Jun 24;15(13):4920. doi: 10.3390/jcm15134920 (PMC13361525; doi:10.3390/jcm15134920)
Supplement: Supplementary file 1 [file jcm-15-04920-s001.zip › jcm-4366700-Supplementary Materials S1 and S2.pdf]

Search Strategy Document

Artificial Intelligence-Based Interventions for Non-Specific Low Back Pain: A Systematic Review and Meta-Analysis

---

S1. Search Dates

Initial Search: March 2025

Updated Search: May 2026

S2. Electronic Database Search Strategies

S2.1 PubMed/MEDLINE (NLM)

Line	Search Strategy
#1	“Artificial Intelligence”[Mesh] OR “Machine Learning”[Mesh] OR “Neural Networks, Computer”[Mesh] OR “Deep Learning”[tiab] OR “machine learning”[tiab] OR “artificial intelligence”[tiab] OR “AI-based”[tiab] OR “AI guided”[tiab]
#2	“Low Back Pain”[Mesh] OR “low back pain”[tiab] OR “nonspecific low back pain”[tiab] OR “non-specific low back pain”[tiab] OR “chronic low back pain”[tiab] OR “LBP”[tiab]
#3	“Randomized Controlled Trial”[Publication Type] OR “randomized controlled trial”[tiab] OR “RCT”[tiab] OR “randomised”[tiab] OR “clinical trial”[tiab]
#4	#1 AND #2 AND #3
#5	Animals [Mesh] NOT (Humans [Mesh] AND Animals [Mesh])
#6	#4 NOT #5

Records Identified: 2,847

S2.2 EMBASE (Elsevier)

Line	Search Strategy
#1	(‘artificial intelligence’:ti,ab OR ‘machine learning’:ti,ab OR ‘deep learning’:ti,ab OR ‘neural network’:ti,ab OR ‘AI-based’:ti,ab OR ‘AI guided’:ti,ab)

Line	Search Strategy
#2	(‘low back pain’:ti,ab OR ‘nonspecific low back pain’:ti,ab OR ‘non-specific low back pain’:ti,ab OR ‘chronic low back pain’:ti,ab OR ‘LBP’:ti,ab)
#3	(‘randomized controlled trial’:ti,ab OR ‘RCT’:ti,ab OR ‘randomised’:ti,ab OR ‘clinical trial’:ti,ab)
#4	#1 AND #2 AND #3
#5	#4 NOT [animals]/lim NOT ([animals]/lim AND [humans]/lim)

Records Identified: 1,923

S2.3 Cochrane Central Register of Controlled Trials (CENTRAL)

Line	Search Strategy
#1	MeSH descriptor: [Artificial Intelligence] explode all trees
#2	MeSH descriptor: [Machine Learning] explode all trees
#3	(“deep learning” OR “machine learning” OR “artificial intelligence” OR “AI-based” OR “AI guided”):ti,ab,kw
#4	MeSH descriptor: [Low Back Pain] explode all trees
#5	(“low back pain” OR “nonspecific low back pain” OR “non-specific low back pain” OR “chronic low back pain” OR “LBP”):ti,ab,kw
#6	#1 OR #2 OR #3
#7	#4 OR #5
#8	#6 AND #7 in Trials

Records Identified: 412

S2.4 Web of Science (Clarivate)

Line	Search Strategy
#1	TS=(“artificial intelligence” OR “machine learning” OR “deep learning” OR “neural network” OR “AI-based” OR “AI guided”)
#2	TS=(“low back pain” OR “nonspecific low back pain” OR “non-specific low back pain” OR “chronic low back pain” OR “LBP”)

Line	Search Strategy
#3	TS=("randomized controlled trial" OR "RCT" OR "randomised" OR "clinical trial")
#4	#1 AND #2 AND #3

Records Identified: 987

S2.5 CINAHL (EBSCO)

Line	Search Strategy
#1	(MH "Artificial Intelligence+") OR (MH "Machine Learning+")
#2	TI("deep learning" OR "machine learning" OR "artificial intelligence" OR "AI-based" OR "AI guided") OR AB("deep learning" OR "machine learning" OR "artificial intelligence" OR "AI-based" OR "AI guided")
#3	(MH "Low Back Pain+")
#4	TI("low back pain" OR "nonspecific low back pain" OR "non-specific low back pain" OR "chronic low back pain" OR "LBP") OR AB("low back pain" OR "nonspecific low back pain" OR "non-specific low back pain" OR "chronic low back pain" OR "LBP")
#5	(MH "Clinical Trials+")
#6	TI("randomized controlled trial" OR "RCT" OR "randomised" OR "clinical trial") OR AB("randomized controlled trial" OR "RCT" OR "randomised" OR "clinical trial")
#7	#1 OR #2
#8	#3 OR #4
#9	#5 OR #6
#10	#7 AND #8 AND #9

Records Identified: 534

S2.6 PEDro (Physiotherapy Evidence Database)

Line	Search Strategy
#1	Abstract & Title: "artificial intelligence" OR "machine learning" OR

Line	Search Strategy
	“deep learning”
#2	Abstract & Title: “low back pain” OR “nonspecific low back pain” OR “chronic low back pain”
#3	Therapy: Exercise AND Problem: Back pain
#4	#1 AND #2
Records Identified: 144	
